# Proteasome inhibition paradoxically degrades gain-of-function mutant p53 R273H in NSCLC and could have therapeutic implications

**DOI:** 10.3389/fonc.2024.1363543

**Published:** 2024-04-10

**Authors:** Eziafa I. Oduah, Susan T. Sharfstein, Nagashree Seetharamu, Steven R. Grossman, Larisa Litovchick

**Affiliations:** ^1^ Duke University School of Medicine, Division of Oncology, Durham, NC, United States; ^2^ Duke Cancer Institute, Durham, NC, United States; ^3^ Department of Internal Medicine, Division of Hematology, Oncology and Palliative Care, Massey Comprehensive Cancer Center and Virginia Commonwealth University, Richmond, VA, United States; ^4^ State University of New York at Albany, College of Nanoscale Science and Engineering, Albany, NY, United States; ^5^ Donald and Barbara Zucker School of Medicine at Hofstra, Division of Medical Oncology and Hematology, Lake Success, NY, United States; ^6^ Keck School of Medicine and USC Norris Comprehensive Cancer Center, University of Southern California, Los Angeles, CA, United States

**Keywords:** NSCLC, proteasome, TP53, mutant p53, proteasome inhibitor

## Abstract

Lung cancer is the leading cause of cancer mortality. Despite therapeutic advances in recent years, new treatment strategies are needed to improve outcomes of lung cancer patients. Mutant p53 is prevalent in lung cancers and drives several hallmarks of cancer through a gain-of-function oncogenic program, and often predicts a poorer prognosis. The oncogenicity of mutant p53 is related to its stability and accumulation in cells by evading degradation by the proteasome. Therefore, destabilization of mutant p53 has been sought as a therapeutic strategy, but so far without clinical success. In this study, we report that proteasome inhibition results in degradation of mutant p53 in non-small cell lung cancer (NSCLC) cell lines bearing the R273H mutant protein and show evidence that this was mediated by hsp70. NSCLC cell lines with the mutant R273H allele demonstrated increased susceptibility and apoptosis to proteasome inhibitors. These data suggest that proteasome inhibitors could have therapeutic implications in some subsets of *TP53* mutated NSCLC.

## 1 Introduction

Despite advances in targeted approaches and immunotherapy, lung cancer remains the leading cause of cancer mortality in the United States and worldwide. The poorer prognosis remains partly because these therapies are not applicable to all patients and are often limited by primary or secondary resistances, highlighting the need for continued search for new therapeutic strategies for patients with non-small cell lung cancer (NSCLC).

*TP53* mutations are prevalent in NSCLC and are harbingers of poorer prognosis and subdued responses to targeted therapies (1, 2). In its wildtype state, p53 is a tumor suppressor whose function is to maintain genome integrity by controlling key cellular processes including cycle arrest, DNA repair, and apoptosis (3, 4). Loss of normal p53 function, occurring through aberrant regulation and/or structural changes, is central to carcinogenesis. Amongst structural changes, somatic mutations are the most common, and result in an abnormal mutant p53 protein with a spectrum of unusual activities. This spectrum of activity could be either due to a loss of tumor suppression function; a dominant negative effect whereby the mutant allele suppresses the remaining wildtype allele; or a gain-of- function (GOF) effect due to acquisition of oncogenic properties via transactivation of pro-oncogenic pathways.

GOF mutations of the *TP53* gene (encoding p53) represent about a third of all p53 genetic alterations in NSCLC. These arise from missense mutations in *TP53* and are characterized by loss of heterozygosity and unusual protein stability, the mechanisms of which are not fully understood. It is well established that the oncogenicity of GOF mutant p53 is related to its stability and excess accumulation in the cancer cell (5–8). In contrast to wild type p53 protein, oncogenic GOF mutant p53 evades degradation by the ubiquitin proteasome system (UPS), resulting in accumulation of the mutant protein to high levels. Therefore, the degradation of oncogenic mutant p53 has been sought as a therapeutic strategy, but so far without clinical success.

We found evidence of paradoxical degradation of GOF mutant p53 by proteasome inhibition. Although a few other reports of this phenomenon are available in the literature, its mechanism has not been completely understood, nor was it sought as a therapeutic strategy directed against GOF mutant p53 (9–11). Since these initial reports, new proteasome inhibitors have become FDA approved for clinical use in certain hematologic malignancies such as multiple myeloma. In this study, we aimed to further understand the mechanism(s) of the oncogenic GOF p53 degradation by proteasome inhibition and its therapeutic potential in this subset of NSCLC.

## 2 Materials and methods

### 2.1 Cell lines and reagents

Cell lines and culture conditions. A549 and H1975 were previously obtained from ATCC and maintained in RPMI-1640 (Gibco). The isogenic H1299 cell line with stably transfected R273H mutant, normal wild type p53 and vector control were a gift from Sumitra Deb (Virginia Commonwealth University) and also maintained in RPMI-1640 (Gibco). RPMI was supplemented with 1% (v/v) Penicillin/Streptomycin (Cat. No 30-002CI, Corning) and 10% (v/v) FBS (Cat. no. S11150, Atlanta Biology). Cells were frequently checked for mycoplasma contamination using PCR assay and/or DAPI staining. For passaging, cells were first washed once with 1X PBS (Corning, Cat# MT21-031-CV) then detached using 0.25% Trypsin/EDTA (Gibco, Cat# 25200-056) followed by re-suspension in fresh growth medium. Cell counting was performed using the Cytosmart automatic cell counter (Corning) and seeded according to experimental requirement in either 10 cm, 6-well or 96-well tissue culture plates. All cell-based assays included at least 3 biological repeats.

### 2.2 Cycloheximide assay

Cells were pretreated with or without 100nm of bortezomib for 18 hours. Cycloheximide was freshly dissolved in DMSO and added to a final concentration of 50mg/ul. Cells were harvested at the indicated time points followed by western blotting as described below.

### 2.3 Western blotting and co-immunoprecipitation

For western blotting, cells were lysed in NP40 lysis buffer [25mM Tris HCl (pH 7.5), 150mM NaCl, 1% NP40], supplemented with protease inhibitors (Roche) for 30 minutes at 4°C and then centrifuged at 14,000g for 20 minutes. Protein concentrations were determined by DC protein assay (BioRad) by measuring absorbances at 750 nm. Protein samples (10-20 µg per well) were denatured by heating in SDS-PAGE loading buffer, resolved using polyacrylamide gels (Thermofisher), transferred to PVDF (Thermofisher) or nitrocellulose membrane (GE Healthcare). Membranes were blocked with LI-COR blocking buffer (LI-COR Biosciences) and probed overnight at 4˚C with primary antibodies (**Supplementary Table 1**) diluted 1:500-1:1,000 in the same buffer. Membranes were washed with TBS-T thrice for 5 min each and then incubated with mouse or rabbit secondary antibodies (1:1,000 dilution; LI-COR antibodies) for 1 hour at room temperature. The blots were developed with LI-COR Image studio software.

For co-immunoprecipitation, cells were lysed as described above. Clarified lysates were incubated overnight at 4°C with 10 µl of Protein A agarose (for antibodies raised in rabbit) or Protein G agarose (for antibodies raised in mouse) and 1 µg anti-p53 antibody (clone FL-393; Cat No. sc-6243, Santa Cruz Biotechnology, Inc.) or anti-hsp70 antibody (Cat No. sc-27, Santa Cruz Biotechnology, Inc.). The beads were then washed 5 times with lysis buffer and immunoprecipitates were eluted by heating in SDS-PAGE loading buffer. Aliquots of IP eluates and cell lysate (input) were resolved by SDS-PAGE on 4-12% gels, transferred to PVDF membrane and probed with specific antibodies.

### 2.4 Annexin V-FOTC/PI apoptosis assay

Silencing of hsp70 was achieved by transiently transfecting cells with Silencer Select predesigned siRNA s194536 (Cat. no. 4392421, Thermofisher Scientific). siRNAs were transfected at a final concentration of 10 nM with lipofectamine RNAiMax (Cat. no. 137781030, ThermoFisher Scientific) for 48 hours and then treated with indicated drugs or vehicle control (DMSO). As a control, the Silencer Select Negative Control siRNA (ThermoFisher Scientific) was used at the same concentration and for the same time.

### 2.5 Cell viability assay

Cell viability was determined using the MTS-based assay Promega Cell Titer Aqueous One (Promega, Cat. No. G3582). Cells were seeded in 96-well plates at a seeding density of 15,000 cells/well. Cells were then treated with indicated drugs and incubated for 24 and 48 hours. Cell staining and quantification of viability were performed according to the manufacturer’s instructions. All experiments were performed in triplicate.

### 2.6 Apoptosis and annexin V-FITC and PI staining assay

Apoptotic cells were detected by an annexin V-FITC/PI apoptosis detection kit (Thermofisher, USA) according to manufacturer’s instruction. After incubation with bortezomib for 24h, cells were trypsinized, washed, and collected (1x10^6^ cells). Cells were then suspended in binding buffer with staining by Annexin-FITC and PI solution for 30 min. Cells were analyzed using a flow cytometer BD FACS Canto II (Franklin Lakes, NJ).

### 2.7 Statistical analysis

Data represented as mean ± SD of at least 3 biological replicates unless indicated otherwise. Unpaired Student’s *t*-test was used to compare differences between groups. When the assumption of Gaussian distribution was not met, a non-parametric Mann–Whitney *U*-test was used for comparisons. For comparisons between more than two groups, a one- or two-way ANOVA was performed. A p value of <0.05 was considered statistically significant. Statistical analysis was performed in Graphpad Prism software.

## 3 Results

### 3.1 Proteasome inhibition results in degradation of mutant but not wildtype p53 in lung cancer cell lines

**Figure 1:**
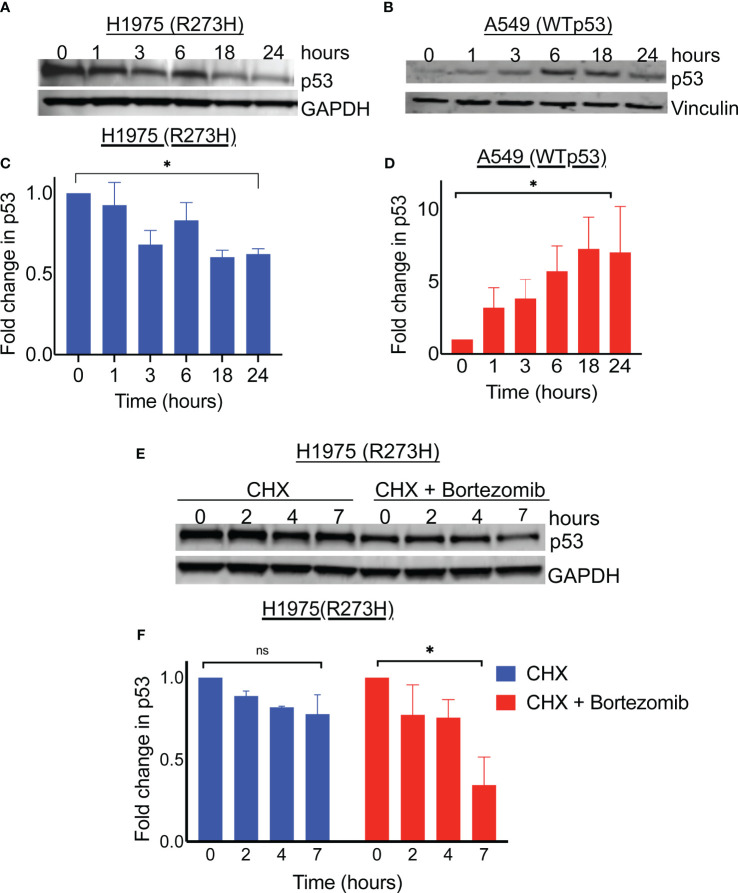
Proteasome inhibition results in degradation of mutant p53 protein. Immunoblot analysis of p53 level in R273H mutant bearing lung cancer cell line (H1975) and wildtype p53 bearing lung cancer cell line (A549) treated with 100nM Bortezomib for indicated timepoints **(A, B)**. The intensity of p53 was determined by densitometry using ImageJ software and expressed relative to control **(C, D)**. **(E)** Lung cancer cell line H1975 (p53R273H) was treated with cycloheximide with and without bortezomib. Cell lysates were obtained at indicated timepoints and immunoblotted with indicated antibodies. **(F)** Detected bands were quantified on image J and plotted relative to 0 time point. Graph shows average of 3 replicates. * = p<0.05, ns = not significant.

We had previously observed that oncogenic mutant p53 was paradoxically depleted when the proteasome inhibitor, MG132, was used as a positive control for ubiquitination (data not shown). We therefore sought out to determine whether the FDA approved proteasome inhibitor, bortezomib, produced a similar effect of degrading the mutant protein. The lung cancer cell line H1975, which harbors the missense mutant p53 R273H was treated with 100nM bortezomib and harvested at different timepoints up to 24 hours, followed by cell lysis and immunoblotting for p53. As shown in **Figures 1A, C**, the level of the detected protein decreased with time following proteasome inhibition. Since the proteasome is the primary degradation machinery for wildtype p53, inhibition of the proteasome is expected to result in an accumulation of the wildtype protein. Therefore, we also evaluated the effect of proteasome inhibition in wildtype p53. For this, we used the lung cancer cell line A549, which harbors wildtype p53. Indeed, an inhibition of the proteasome with bortezomib resulted in accumulation of wildtype p53 in a time dependent manner as expected (**Figures 1B, D**). These results suggested that the depletion of GOF mutant p53, induced by proteasome inhibition, was a phenomenon specific to the mutated protein, and not the wildtype p53 protein.

To evaluate whether the depletion of mutant p53 in the H1975 cells was by a degradation mechanism, we performed a cycloheximide chase assay and the degree of protein decay overtime was determined by immunoblotting. As shown in **Figures 1E, F**, the mutant p53 levels decreased with time in the cells treated with bortezomib followed by cycloheximide, compared to the cells treated with cycloheximide alone. Since GOF mutant p53 degradation was occurring in the context of proteasome blockade, we suspected that diversion to an alternative degradation pathway was at play. Autophagy is one of the protein degradation mechanisms in mammalian cells and was previously implicated in mutant p53 degradation mediated by heat shock protein 70 (Hsp70) (12, 13). In support of autophagy induction, we observed LC3B conversion co-incidental to increased levels of Hsp70 upon the treatment of H1975 cells with bortezomib (**Supplementary Figure 1**). In their report, Choudhury et al. also described an autophagic degradation of the mutant p53 upon proteasome inhibition, however the role of Hsp70 was not explored (11). Therefore, we focused our studies on the potential role of Hsp70 played in this degradation of mutant p53 by proteasome inhibition.

### 3.2 Hsp70 is involved in the degradation of mutant p53 by proteasome inhibition in lung cancer

**Figure 2:**
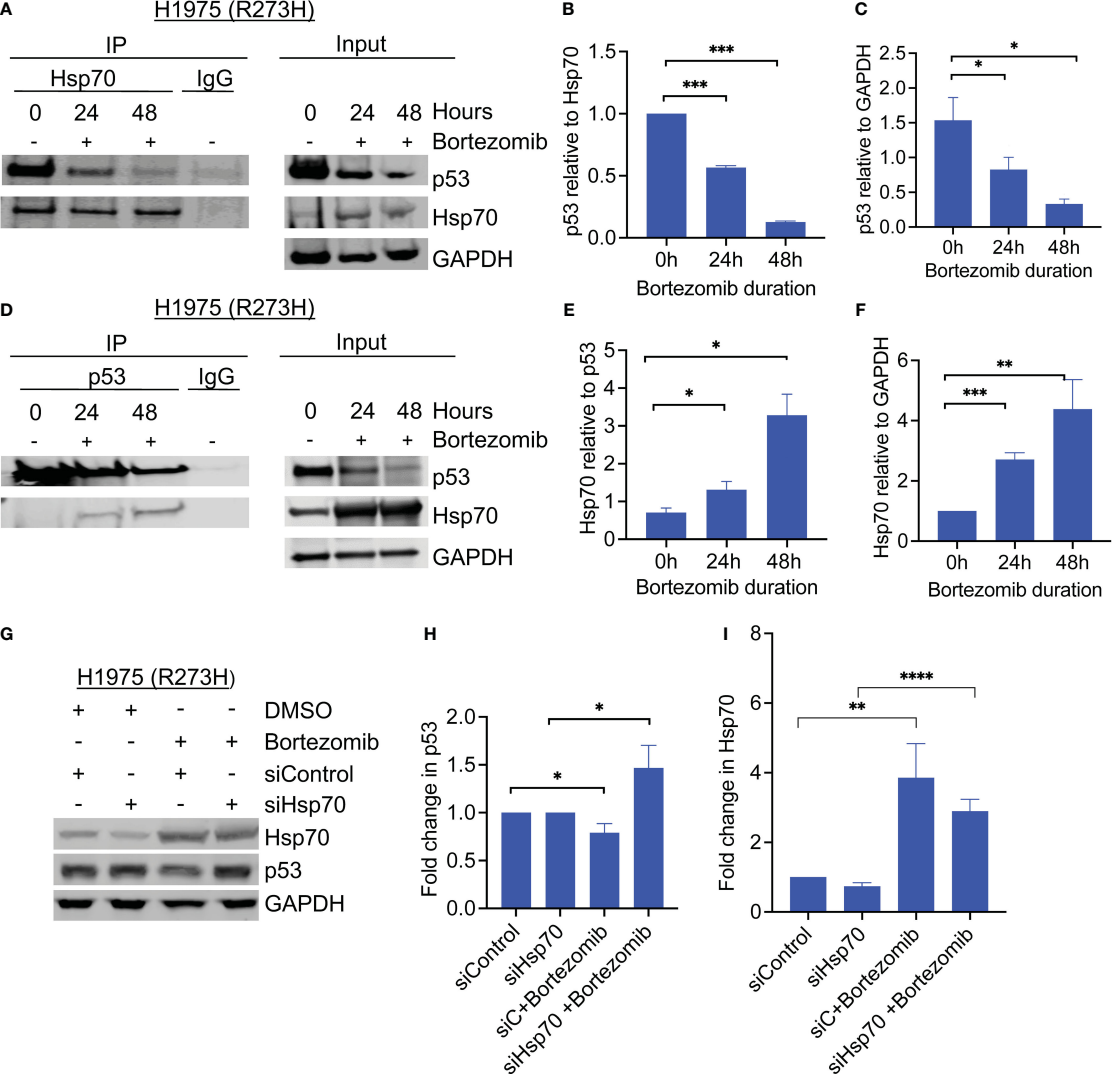
Hsp70 is involved in mutant p53 degradation by bortezomib. **(A)** Co-immunoprecipitation of Hsp70 was performed from H1975 (p53R273H) cells before and after treatment with bortezomib, and samples were immunoblotted with the indicated antibodies. **(B, C)** Detected bands were quantified by densitometry using ImageJ software. The graphs show changes in the co-immunoprecipitated proteins relative to each other and to GAPDH. **(D)** Same as A, only with p53 co-immunoprecipitation. **(E, F).** Detected bands were quantified by densitometry using ImageJ software. The graphs show changes in the co-immunoprecipitated proteins relative to each other and to GAPDH in the input. **(G)** Cells were transfected with control or hsp70-specific siRNA and treated with bortezomib for 24 hours. Hsp70 knockdown rescued bortezomib-induced degradation of mutant p53. **(H)** Quantitative analysis of detected p53 bands was performed using ImageJ software, normalized to GAPDH, and plotted relative to control. **(I)** Quantitative analysis of detected Hsp70 bands was performed using ImageJ software, normalized to GAPDH, and plotted relative to control Graphs show average of 3 biological replicates. Error bars indicate standard deviation. N=3, * p <0.05, ** p<0.01, *** p<0.001, **** p<0.0001.

Hsp70 is a member of the heat shock chaperone family of proteins that are involved in complex cellular processes including the regulation of misfolded proteins (12). It was previously reported that Hsp70 mediated autophagic degradation of mutant p53 under conditions of cellular stress (13). Since the degradation of the mutant p53 by Hsp70 involves the binding of these proteins, their interaction was evaluated before and after the treatment with bortezomib. Hsp70 was first immunoprecipitated before and after treatment with bortezomib, followed by western blotting for p53 and Hsp70. As shown in **Figures 2A–C**, Hsp70 was bound to mutant p53 both before and after treatment with bortezomib, however, the level of co-immunoprecipitated mutant p53 decreased with time, likely due to decreasing levels of p53 in the input. When the reverse co-immunoprecipitation was performed, in which the mutant p53 protein was first pulled down, followed by western blotting for p53 and Hsp70; we found that more Hsp70 co-immunoprecipitated with mutant p53 after the treatment with bortezomib. Interestingly, the relative abundance of Hsp70 that co-immunoprecipitated with mutant p53 was increased after the treatment, even though significantly less p53 was pulled down (**Figures 2D–F**). These findings showed that proteasome inhibition induced elevated levels of Hsp70, coincidental with decreased levels of p53, suggesting a role for Hsp70-mediated degradation of the mutant p53.

To determine whether Hsp70 mediated the degradation of mutant p53 by proteasome inhibition, we performed siRNA-mediated knockdown of Hsp70 in the same GOF p53 lung cancer cell line H1975. Control-RNA or Hsp70 siRNA were transiently transfected into the cells, followed by incubation with or without bortezomib. Immunoblotting of the cell lysates showed that siRNA-mediated knockdown of Hsp70 stabilized mutant p53 levels and rescued the effect of bortezomib (**Figures 2G–I**). Together, these findings confirm the role of Hsp70 in mediating the degradation of mutant p53 under conditions of proteasome inhibition.

### 3.3 Gain of function p53 R273H lung cancer cell lines demonstrate enhanced sensitivity to proteasome inhibition

Since GOF mutant p53 accumulation is important for its oncogenic activities and survival of the cancer cells, we hypothesized that the observed phenomenon of GOF p53 degradation by proteasome inhibitors could have functional implications for their therapeutic use in GOF p53 mutant NSCLC.

**Figure 3:**
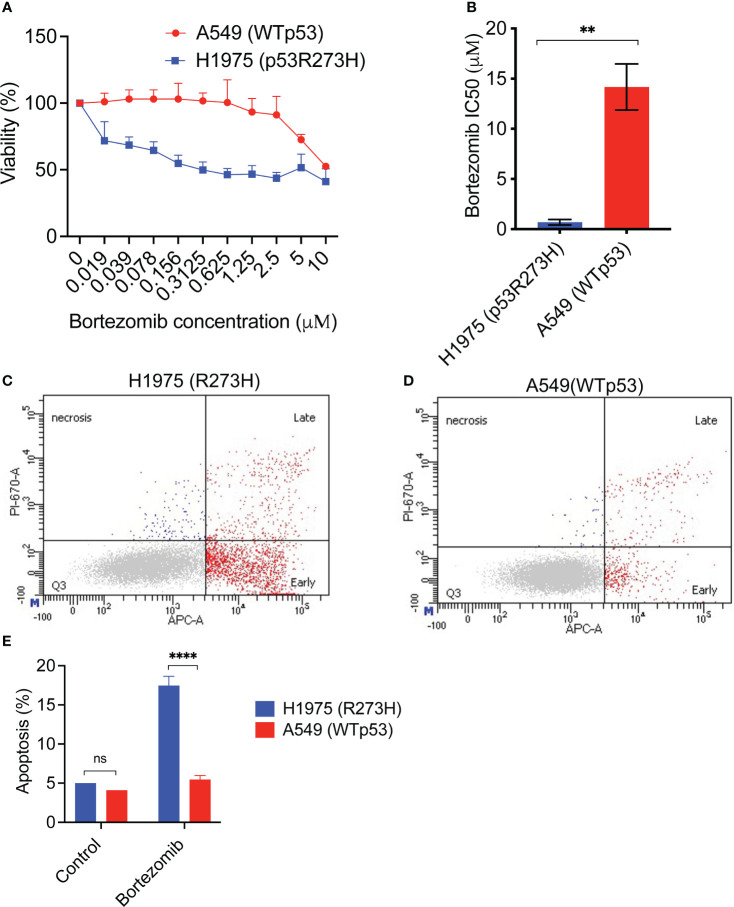
An NSCLC cell line with mutant p53 R273H demonstrates increased sensitivity and apoptotic response to bortezomib. **(A)** The lung cancer cell lines H1975 (R273H) and A549 (WTp53) were treated with increasing doses of bortezomib for 48 hours, followed by cell viability assay. The graph shows average of 3 replicates +/- SD. **(B)** The IC50 for bortezomib was calculated using GraphPad Prism software. The graph shows average of at least 3 independent IC50 determinations +/-SD. **(C, D)** H1975 (R273H) and A549 (WTp53) were treated with bortezomib for 24 hours. Cells were harvested and evaluated for apoptosis by Annexin V stain followed by flow cytometry. Representative flow cytometry data are shown. **(E)** Quantification of the data in **(C)** and **(D)**. The graph shows the total apoptosis in bortezomib treated cells compared to untreated control, average +/-SD. N=3, ** p <0.01, **** p<0.0001, ns =not significant.

To test this hypothesis, we first analyzed the cytotoxic effect of bortezomib using the human lung cancer line H1975 which harbored the mutant p53R273H, contrasted against the wildtype p53 human lung cancer cell line A549. The cells were the treated with increasing concentrations of bortezomib for 24 hours followed by cell viability assays. The IC50 of bortezomib was then calculated for the cell lines. As shown in **Figures 3A, B**, H1975 (p53 R273H) cell line was more sensitive to bortezomib and had a lower IC50 compared to A549 (wildtype p53).

Next, we determined the apoptotic response of the two cell lines to bortezomib. After treatment with bortezomib for 24 hours, the cells were stained with Annexin V followed by flow cytometry. Interestingly, the gain-of-function mutant p53 cell line H1975 (R273H) had a statistically significant increased apoptotic response to bortezomib compared to the wildtype p53 cell line (**Figures 3C–E**).

**Figure 4:**
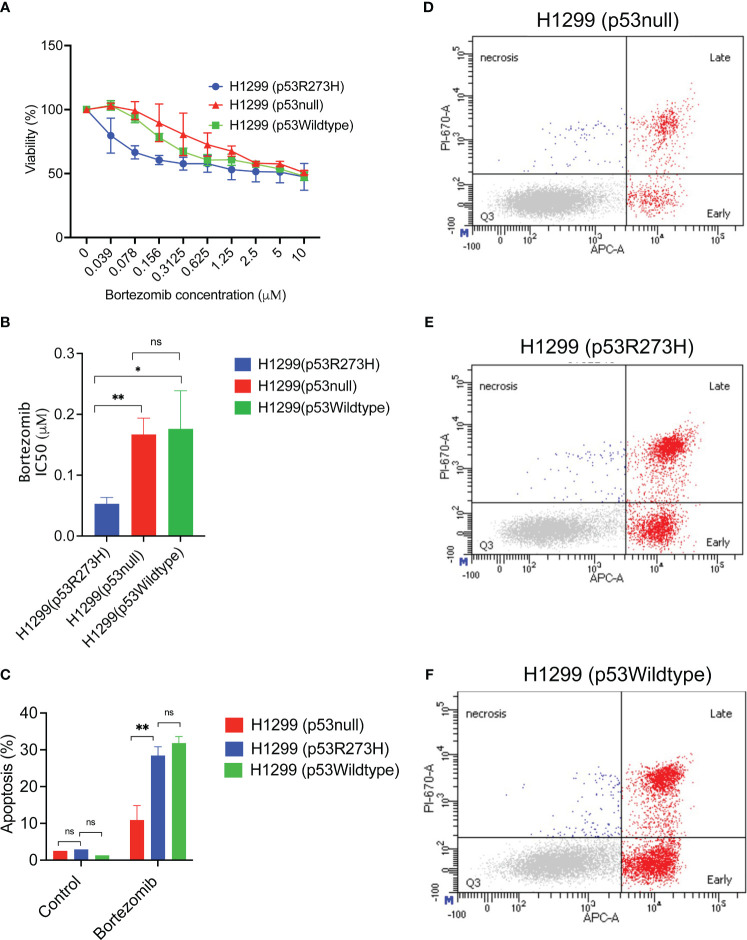
Increased sensitivity and apoptotic response to bortezomib could be due to mutant p53 R273H status. **(A)** The isogenic cell line set H1299 stably transfected to express vector control, the mutant p53 R273H or wildtype p53 rescue were treated with bortezomib for 48 hours, followed by cell viability assay. The graph shows average of at least 3 biological replicates +/- SD. **(B)** The IC50 for bortezomib was calculated using GraphPad Prism software. The graph shows average of at least 3 biological replicates +/-SD. **(C)** The H1299 isogenic cell line set (p53R273H, p53null, p53wildtype) were treated with bortezomib followed by annexin V stain and flow cytometry. The graph shows total apoptosis in bortezomib treated cells and untreated controls, average +/-SD. **(D–F)** Representative flow cytometry data are shown for indicated cell lines. (N=3, ** p<0.01, * p<0.05, ns =not significant.

To determine whether the enhanced susceptibility to bortezomib was due to the presence of p53 R273H, we used an isogenic cell line set in which the p53 null human NSCLC cell line H1299 was stably transfected to either express a null vector control, the p53 R273H mutant or a wildtype p53 rescue construct. Consistent with the observation with lung cancer cell lines expressing the mutant or the wild type p53 (**Figure 1**), the treatment with bortezomib resulted in degradation of the ectopically expressed p53R273H and accumulation of the wild type p53 protein (**Supplementary Figure 2**). Next, the isogenic cell lines with mutant p53 R273H, wildtype p53 or a null control were treated with increasing concentrations of bortezomib followed by cell viability assay. The IC50 of bortezomib was then calculated for these cell lines. As shown in **Figures 4A, B**, the cell line ectopically expressing the p53 R273H mutant was sensitive to bortezomib at lower doses and had a lower IC50 compared to the control or wildtype p53 rescued cell lines.

Next, the apoptotic response to bortezomib was also evaluated in the same cell lines. After treatment with bortezomib, the cells were stained with Annexin V followed by flow cytometry. The p53 R273H cell line demonstrated statistically significant increased apoptosis compared to the null vector control, but similar to the wildtype p53 rescued cell line control. (**Figures 4C–F**). Interestingly, compared to the mutant p53 R273H, the normal wildtype p53 had an increased fraction of the early apoptotic cells and a decreased late apoptosis, although this trend was not statistically significant (**Supplementary Figure 3**). This could explain the differences in sensitivity, since the early apoptosis is reversible and the cells in this state can continue DNA synthesis (14). Together, these results suggested that the presence of the mutant p53 R273H may play a role in determining sensitivity to bortezomib.

## 4 Discussion

The role of Hsp70 in the degradation of GOF mutant p53 either by the proteasome or autophagy is well documented. Our mechanistic studies support a role for Hsp70 in the degradation of mutant p53 in bortezomib-treated cells. This conclusion is supported by evidence of the interaction between the mutant p53 and Hsp70 both in the absence and in the presence of bortezomib, and by the rescue of mutant p53 degradation by Hsp70 knockdown. Since proteasomal degradation is inhibited by bortezomib, it is reasonable that Hsp70-mediated degradation of the mutant p53 is accomplished through an autophagic degradation pathway. Indeed, a detailed study of the autophagic degradation of mutant p53 by the proteasome inhibitor, MG132, was previously reported by Choudhury et al. (11). However, the role of Hsp70 was not investigated in that study. In this report, we add to the body of evidence supporting the degradation of mutant p53 induced by proteasome inhibition, and the role for Hsp70 in this process. It is noteworthy that bortezomib had no impact on the level of Hsp90 (data not shown). Since Hsp90 binds mutant p53 to block the E3 ligase activity of MDM2 and CHIP, this suggested that the degradation of mutant p53 by bortezomib was unrelated to these E3 ligase activities and was not investigated further (15). On the other hand, it is also possible that the induction of Hsp70 disrupts the homeostasis of the heat shock protein complexes with mutant p53, liberating it for degradation.

To our knowledge, this work also represents a first attempt to investigate the functional impact of proteasome inhibition specific to a *TP53* mutation in NSCLC. The cumulative evidence of apoptosis induction and increased susceptibility in the R273H p53 mutant raises the possibility that proteasome inhibitors could be used to therapeutically target this subset of NSCLC. Interestingly, bortezomib significantly induced more apoptosis in the mutant p53 lung cancer cell line H1975 (R273H) compared to the wildtype p53 lung cancer cell line A549. In the isogenic cell line panel, we demonstrated that the presence of the mutant R273H led to increased susceptibility to bortezomib and significantly increased apoptosis in the mutant R273H compared to the p53 null control or the wildtype p53-rescued cells. However, a similar apoptotic response seen between the mutant p53 R273H and the wildtype p53 rescued cell lines indicates that there is a need to further investigate the mechanism of increased sensitivity of the mutant p53R273H cells. One possible explanation could be an increased rate of a potentially reversible early apoptosis in the wildtype p53 compared to the p53R273H-rescued cell line upon bortezomib treatment. In addition to the degradation of the mutant p53 protein likely playing a significant role in the increased susceptibility to proteasome inhibition, it is possible that other factors are contributory and will be investigated in further studies.

In the present report, we have focused on an endogenously expressed R273H mutant p53 in a lung cancer cell line and validated these findings as attributable to the R273H mutant p53 protein using an ectopically expressed R273H in a p53 null lung cancer cell line. R273H is a known hotspot mutant p53 with demonstrated gain-of-function properties. Although it represents only a subset of mutant p53, these findings could have clinical utility in this subset of patients. In addition, the significant differences between the p53 null and R273H gain-of-function p53 indicates that this effect may be more beneficial in gain of function p53 mutants over loss of function p53 mutants that are functionally p53-null. In cancer, the normal p53 function is often lost in due to regulatory dysfunction, even when the wildtype protein is retained. However, more studies are necessary to determine the generalizability of these findings in other types of GOF p53 mutations in NSCLC.

The potential to repurpose proteasome inhibitors in lung cancer is limited by prior studies of bortezomib in unselected patients with NSCLC. We previously reviewed earlier clinical trials of proteasome inhibitors in lung cancer (16). These trials did not select nor stratify patients by *TP53* mutation status and yielded lower response rates than would be expected. The *TP53* mutation status of most patients was not known since tumor sequencing was not yet broadly applied to lung cancer during the time of those studies. However, anecdotal evidence identified some super-responders to proteasome inhibition, some of whom had *TP53* mutations (16). It may be that the lower response rates in these trials were in part due to lack of appropriate patient selection. Since oncogenic missense p53 mutants comprise only about 20-30% of non-small cell lung cancers (NSCLC), it is possible that a dilution of the effect could be at play.

Our findings, however, do not currently indicate whether *TP53* mutation will predict response to proteasome inhibition in lung or other cancers. It is unlikely that proteasome inhibitor monotherapy will have significant therapeutic yield but combination strategies with mechanistically synergistic drug combinations may be beneficial. Such strategies may have significant therapeutic implications in *TP53* co-mutated NSCLC and will be determined in further studies.

## Data availability statement

The raw data supporting the conclusions of this article will be made available by the authors, without undue reservation.

## Ethics statement

Ethical approval was not required for the studies on humans in accordance with the local legislation and institutional requirements because only commercially available established cell lines were used.

## Author contributions

EO: Conceptualization, Data curation, Formal analysis, Funding acquisition, Investigation, Methodology, Writing – original draft, Writing – review & editing, Resources. SS: Methodology, Writing – review & editing, Formal analysis, Supervision. NS: Writing – review & editing. SG: Methodology, Supervision, Writing – review & editing, Resources, Validation. LL: Methodology, Writing – review & editing, Resources, Supervision, Validation.
