# Supplementary material for: Proteasome inhibition paradoxically degrades gain-of-function mutant p53 R273H in NSCLC and could have therapeutic implications
Source: Front Oncol. 2024 Apr 10;14:1363543. doi: 10.3389/fonc.2024.1363543 (PMC11039826; doi:10.3389/fonc.2024.1363543)
Supplement: Supplementary file 1 [file Table_1.docx]

Supplementary Material

# Table 1. List of commercially available antibodies

| Protein | Source | Vendor (Catalog No.) |
| --- | --- | --- |
| p53 | Mouse | Santa Cruz (sc-126) |
| p53 | Rabbit | Santa Cruz (sc-6243) |
| Hsp70 | Mouse | Santa Cruz (sc-27) |
| LC3B (D11) XP | Rabbit | Cell Signaling (3868) |
| Hsp70 | Rabbit | Cell Signaling (4872) |
| Vinculin | Rabbit | Sigma (V9131) |
| GAPDH | Mouse | Millipore (Mab37) |
| GFP | Mouse | Santa Cruz (sc-9996) |
| IRDye 680RD | Goat anti-Rabbit | LI-COR 92668071 |
| IRDye 680RD | Goat anti-Mouse | LI-COR 926-68070 |

**
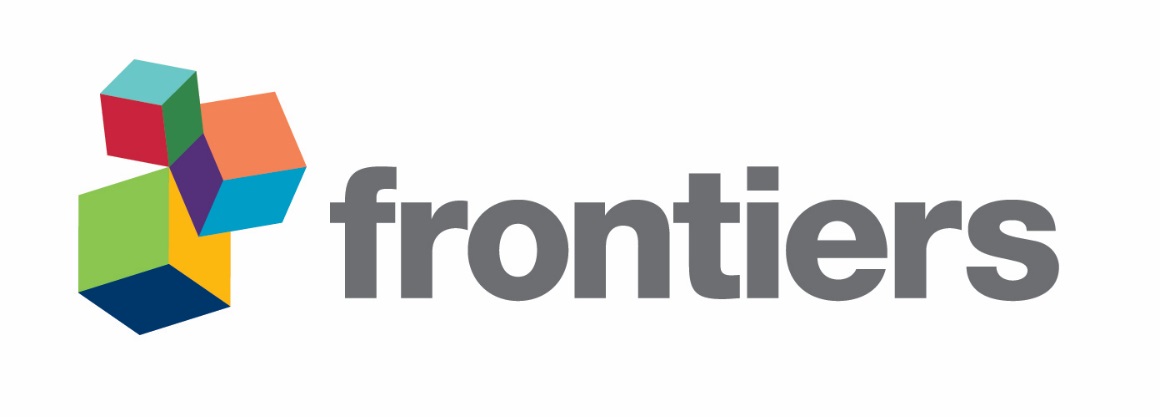
**
