# Supplementary material for: Proteasome inhibition paradoxically degrades gain-of-function mutant p53 R273H in NSCLC and could have therapeutic implications
Source: Front Oncol. 2024 Apr 10;14:1363543. doi: 10.3389/fonc.2024.1363543 (PMC11039826; doi:10.3389/fonc.2024.1363543)
Supplement: Supplementary file 2 [file Presentation_1.pptx]

## Slide 1
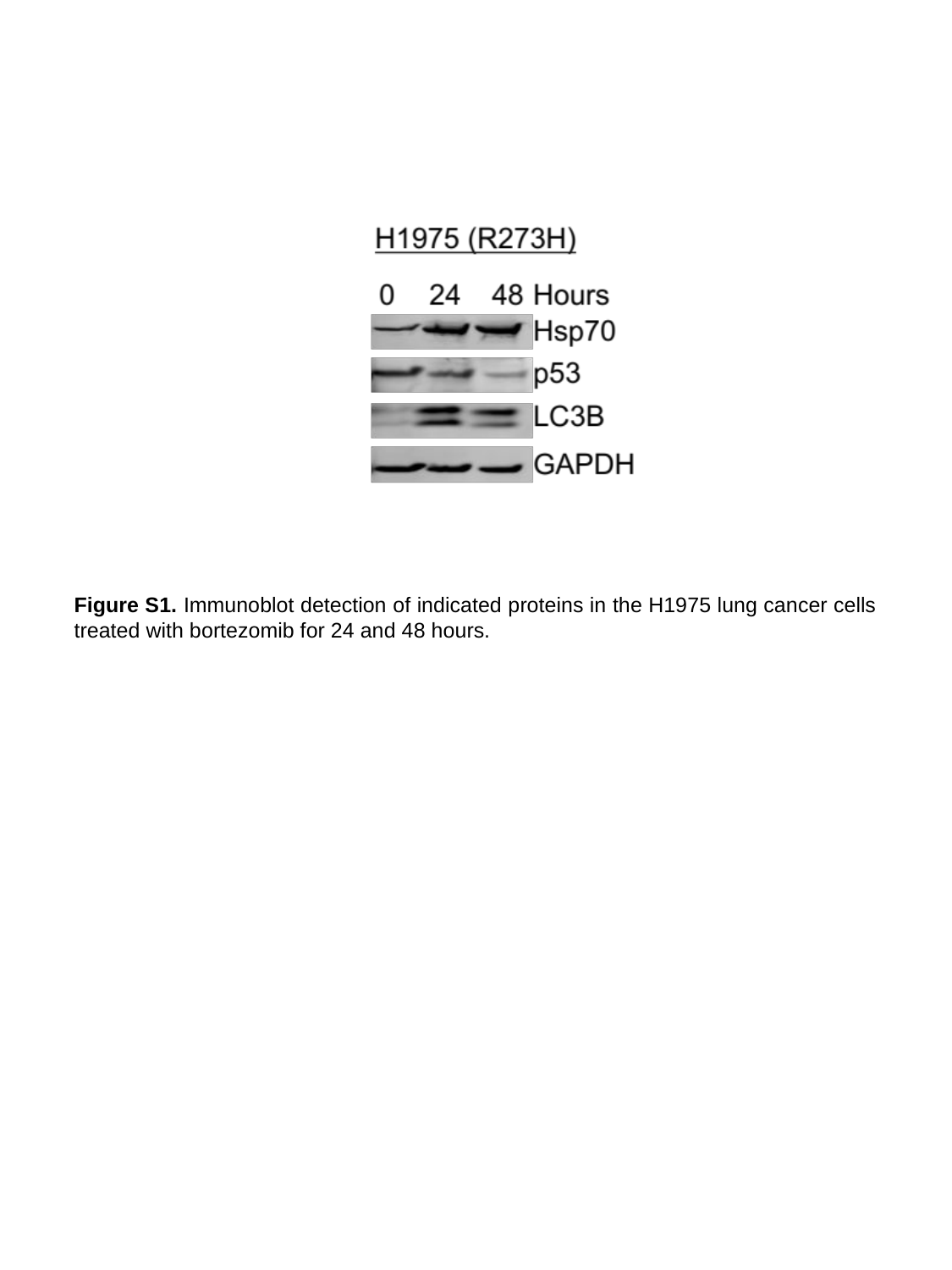

Figure S1. Immunoblot detection of indicated proteins in the H1975 lung cancer cells treated with bortezomib for 24 and 48 hours.

## Slide 2
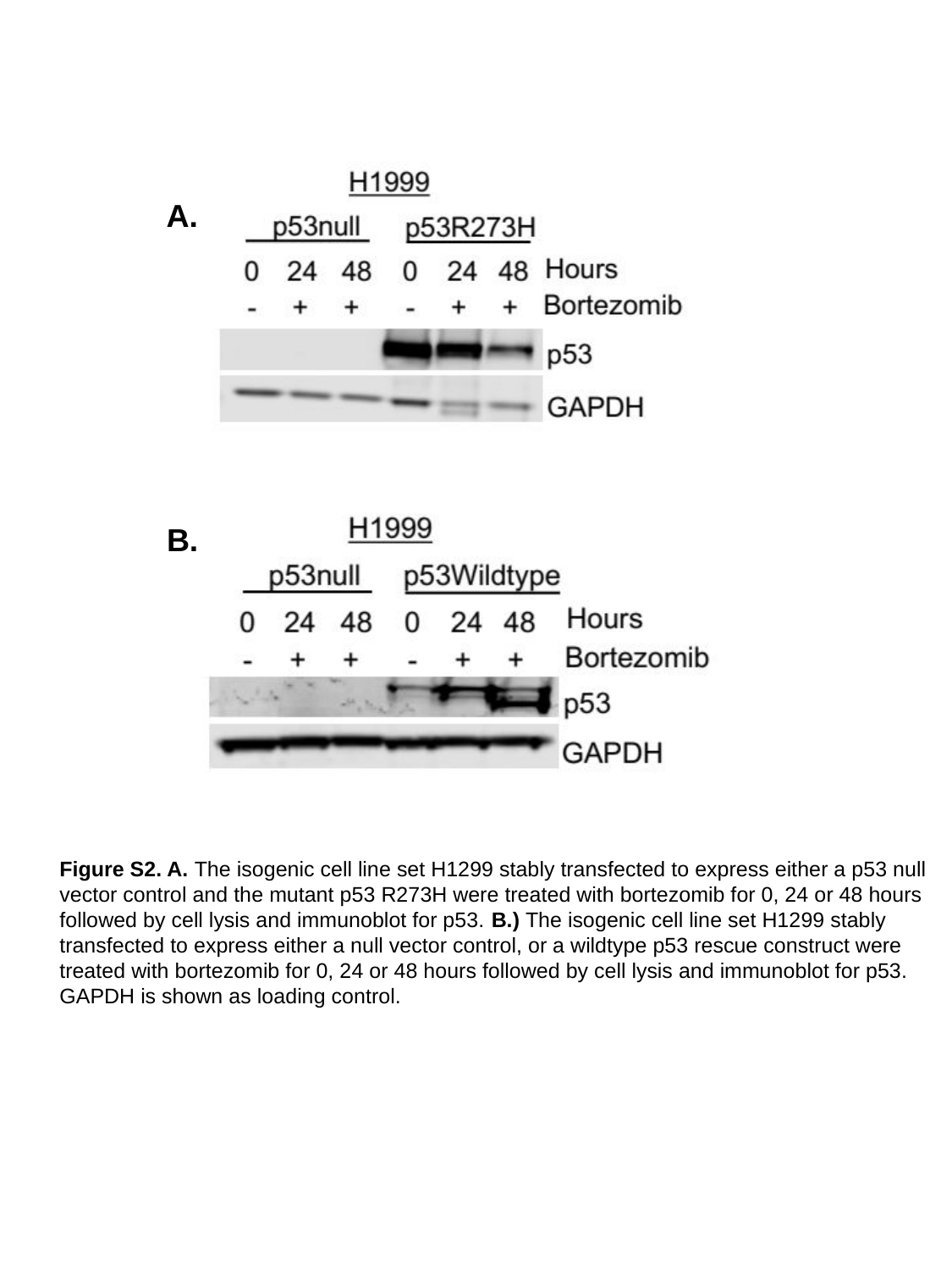

A.
B.
Figure S2. A. The isogenic cell line set H1299 stably transfected to express either a p53 null vector control and the mutant p53 R273H were treated with bortezomib for 0, 24 or 48 hours followed by cell lysis and immunoblot for p53. B.) The isogenic cell line set H1299 stably transfected to express either a null vector control, or a wildtype p53 rescue construct were treated with bortezomib for 0, 24 or 48 hours followed by cell lysis and immunoblot for p53. GAPDH is shown as loading control.

## Slide 3
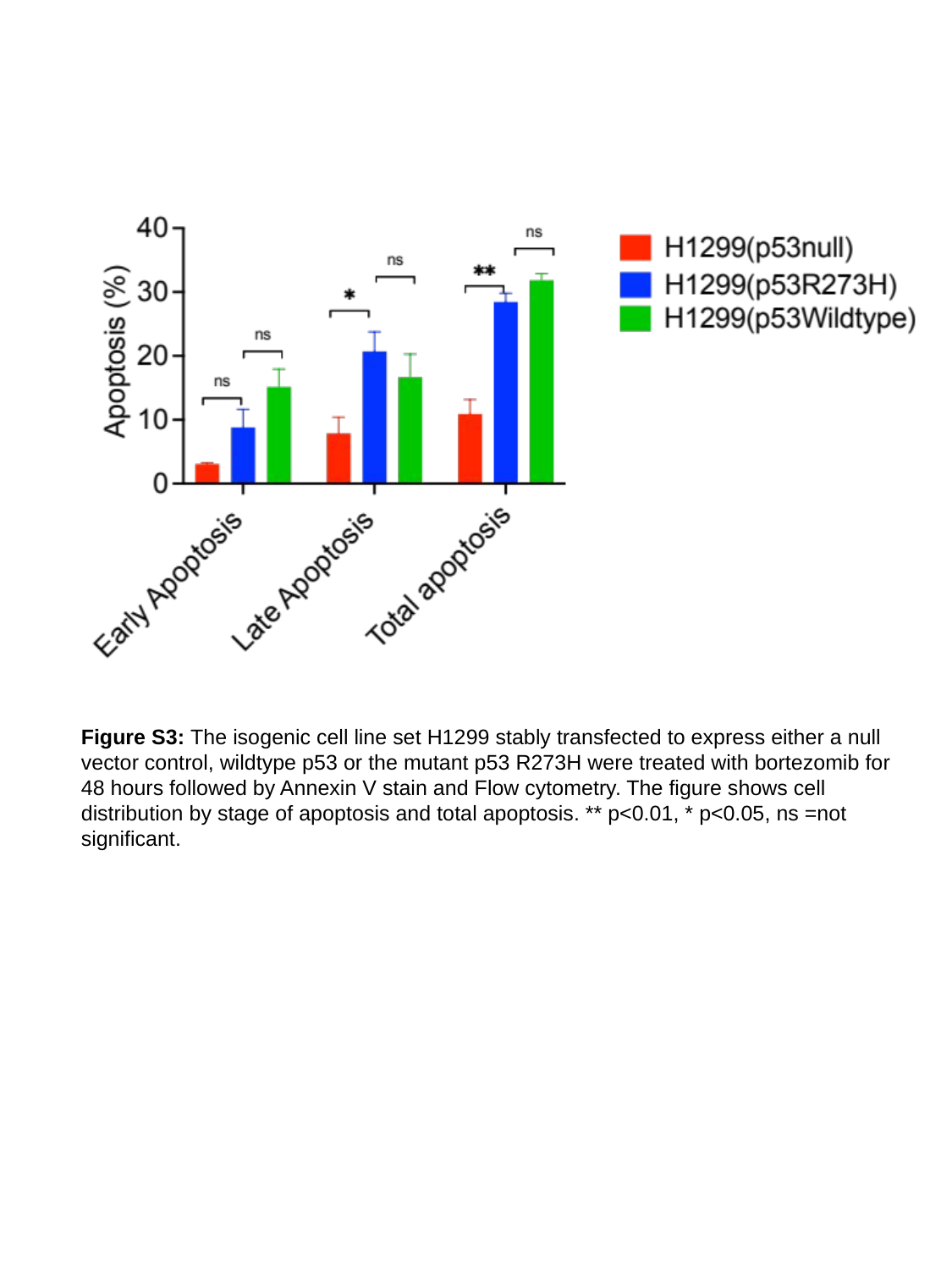

Figure S3: The isogenic cell line set H1299 stably transfected to express either a null vector control, wildtype p53 or the mutant p53 R273H were treated with bortezomib for 48 hours followed by Annexin V stain and Flow cytometry. The figure shows cell distribution by stage of apoptosis and total apoptosis. ** p<0.01, * p<0.05, ns =not significant.
